# Use of Granger causality analysis and artificial spike trains to examine pause coding in Purkinje cell spike activity related to rhythmic licking

**DOI:** 10.1186/1471-2202-12-S1-P143

**Published:** 2011-07-18

**Authors:** Selva K Maran, Ying Cao, Mukesh Dhamala, Detlef Heck, Dieter Jaeger

**Affiliations:** 1 Department of Biology, Emory University, Atlanta, Georgia, 30322, USA; 2 Department of Anatomy and Neurobiology, UTHSC, Memphis, Tennessee, 38163, USA; 3 Department of Physics and Astronomy, GSU, Atlanta, Georgia, 30303, USA

## 

**Figure 1:**
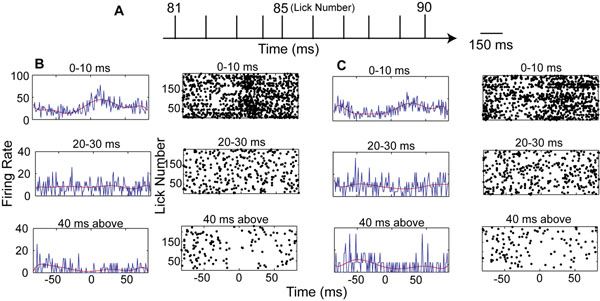
Peri-lick spike histogram broken up by ISI duration. A) The schematic showing the regular lick occurrence with an average lickinterval around 160ms. B) Peri-lick spike histogram for a Purkinje cell recording. The time at which the tongue touches the lick spout is considered time zero. Right column, each dot corresponds to starting time (first spike) of an interspike interval. Left column, the blue trace is the spike rate calculated per millisecond bin. The red trace is polynomial fit for the spike rate. C) Same as (B) but for artificial spike train matched to the Purkinje cell recording with a gamma distribution.

Cerebellar Purkinje cells in mice show strong activity modulation related to rhythmic licking behavior [1]. Here we examine whether this modulation may preferentially be related to long inter-spike intervals (ISIs), i.e. pauses in spike activity. A preferential use of spike pauses for event coding of Purkinje cells has previously been suggested [2,3]. We analyzed the lick modulation of Purkinje cell spike trains by several methods. First, we used peri-lick time histograms constructed separately by spikes initiating ISIs of different duration (Fig. 1B,1C). We found that short and long ISIs showed lick modulation, but licks of intermediate duration did not. Next we conducted a wavelet-based frequency resolved Granger causality analysis [4] to determine whether ISIs of different duration were caused by licks and/or were causal in the control of lick timing. We found there was a peak at 6 Hz from lick to spike in 12 cells, and a smaller peak also at 6 Hz from spike to lick in 5 out of those 12 cells. The peaks for plots (scaled to a possible maximum of 1.0) ranged from 0.1 to 0.8 for lick to spike causality and from 0.1 to 0.3 for spike to lick causality. Again, we found that short (0-10 ms) and long (40 ms and above) were preferentially involved in lick coding. To better understand the ISI duration dependence of lick coding we constructed controlled artificial spike trains from gamma ISI distributions [5], each matching slow spontaneous and lick-triggered spike rate fluctuations. The surrogate data show the same results as the biological ones in terms of preferential short and long ISI involvement in lick modulation. However, causality from spike data to licks was not observed in the artificial data. From these results we infer that any relation between specific ISI durations and lick modulation is due to the statistical properties of rate modulated spike trains, and can be mimicked by a simple gamma - distributed process. We also found that even the spike of single Purkinje cells can be predictive of lick events, suggesting Purkinje cell activity is involved in the control of lick timing.
